# Associated disease risk from the introduced generalist pathogen *Sphaerothecum destruens*: management and policy implications

**DOI:** 10.1017/S003118201600072X

**Published:** 2016-05-24

**Authors:** DEMETRA ANDREOU, RODOLPHE ELIE GOZLAN

**Affiliations:** 1 Faculty of Science and Technology, Bournemouth University, Fern Barrow, Poole, Dorset, BH12 5BB, UK; 2 Institut de Recherche pour le Développement UMR 207 IRD, CNRS 7208-MNHN-UPMC, Muséum National d'HistoireNaturelle, 45 Rue cuvier, 75005 Paris Cedex, France

**Keywords:** aquatic ecosystems, biodiversity threat, topmouth gudgeon, *Pseudorasbora parva*, disease emergence, Europe

## Abstract

The rosette agent *Sphaerothecum destruens* is a novel pathogen, which is currently believed to have been introduced into Europe along with the introduction of the invasive fish topmouth gudgeon *Pseudorasbora parva* (Temminck & Schlegel, 1846). Its close association with *P. parva* and its wide host species range and associated host mortalities, highlight this parasite as a potential source of disease emergence in European fish species. Here, using a meta-analysis of the reported *S. destruens* prevalence across all reported susceptible hosts species; we calculated host-specificity providing support that *S. destruens* is a true generalist. We have applied all the available information on *S. destruens* and host-range to an established framework for risk-assessing non-native parasites to evaluate the risks posed by *S. destruens* and discuss the next steps to manage and prevent disease emergence of this generalist parasite.

## INTRODUCTION

Generalist parasites can infect a wide range of hosts with varying severities; some hosts can be infected but not support the reproduction of the parasite, others can support limited reproduction, whilst, in some hosts the parasite can maximize its reproductive output (Holmes, 1979). The potential host range of a parasite is dictated by physiological, behavioural and ecological attributes of the host that determine the ability of a particular parasite to infect and complete its life cycle (Solter and Maddox, 1998). A genetic basis for potential host suitability is suggested by parasites that are more likely to infect hosts phylogenetically close to their existing ones (Poulin, 2007). Due to existing barriers to dispersal, observed host ranges of parasites often represent only a subset of potential hosts (Perlman and Jaenike, 2003). The process of host translocation in new geographical areas or range expansion by an existing host, allows increased opportunities for parasites to expand their range of potential hosts (Poulin, 2007).

The rosette agent *Sphaerothecum destruens* is a multi-host parasite, which experimental studies have shown to be able to infect a number of salmonid and cyprinid species at varying levels (Arkush *et al*. 1998; Andreou *et al*. 2012). The discovery of *S. destruens* associated with an invasive reservoir host, the topmouth gudgeon *Pseudorasbora parva* (Temminck & Schlegel, 1846), increased the parasite's known and potential species range (Gozlan *et al*. 2005, 2009). The parasite has now been found in established wild populations of a range of fish species in Europe including several of high IUCN (International Union for Conservation of Nature) conservation status (Andreou *et al*. 2011; Ercan *et al*. 2015). In experimental studies, infection with *S. destruens* has also confirmed a high level of mortalities in a range of cyprinid species (Gozlan *et al*. 2005; Andreou *et al*. 2012); proving to be a valuable tool for determining the potential host range of and providing important epidemiological information to model disease emergence (Alshorbaji *et al*. 2015).

As a generalist pathogen, *S. destruens* can infect a range of host species (*n* = 14 up to date) and due to its close association with the invasive *P. parva*, which acts as a healthy reservoir host of *S. destruens* and has rapidly invaded a wide range of ecosystems ranging from Eurasia to the north Africa (Gozlan *et al*. 2010), a risk assessment of *S. destruens*’ potential risk of emergence needs to be established (Copp *et al*. 2009). Here, we aim at (1) calculating the specificity index of *S. destruens* and (2) use all the available information on *S. destruens* and its hosts’ range to evaluate the risk associated with its introduction and make management and prevention recommendations to guide national management agencies and policy makers.

## MATERIAL AND METHODS

### Host specificity index for *S. destruens*

The specificity index (STD) proposed by Poulin and Mouillot (2003) measures the average taxonomic distinctness of a parasite's host species. The specificity index was calculated for *S. destruens* by performing the following steps: (a) *S. destruens* host species were placed within a taxonomic hierarchy using the Linnean classification; (b) the number of steps taken in order to reach a taxon common to two host species were calculated for all possible species pairs; (c) the number of steps was averaged across all species pairs. Step lengths between each hierarchical level were given the equal value of one. The index was calculated using the formula by Poulin and Mouillot (2003):

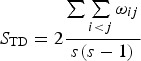

where *s* is the parasite's number of host species, the double summation is over the set (*i* = 1, …*s*; *j* = 1,…*s*, such that *i* < *j*), and *ω*_*ij*_ is the number of taxonomic steps needed to reach a common taxonomic node between host species *i* and *j*. The maximum value that the index STD can reach is five (when using the five taxonomic levels of genus, family, order, class and phylum). The lowest value STD can reach is one and this occurs when all host species share the same genus. A measure of the taxonomic structure of the host species can be obtained by calculating the variance in taxonomic distinctness (Poulin and Mouillot, 2003):

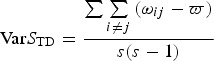

where *ɷ* is the mean taxonomic distinctness or STD. The fish taxonomy proposed by Nelson (1994) was used in calculating the STD for *S. destruens*.

### Evaluating the risk posed by *S. destruens*

We have used an established framework to assess the risk posed by *S. destruens* as described in Williams *et al*. (2013). We considered whether it is possible to manage the spread of *S. destruens*, a criterion central to the OIE (World Organisation for Animal Health) definition underpinning the list of notifiable infectious diseases and determine the potential hazard posed by *S. destruens* using the established risk assessment for managing non-native parasites (Williams *et al*. 2013). We used the case of *S. destruens* and its healthy host *P. parva* in England and Wales as a case study for calculating potential hazard. England and Wales were selected as the case study due to extensive protocols being in place to detect and control the spread of *P. parva*.

## RESULTS

**Fig. 1.**
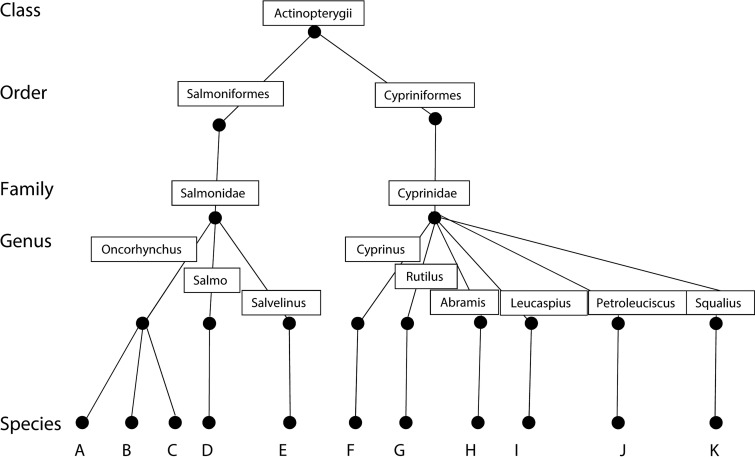
Hierarchical taxonomic tree for all currently known hosts (*n* = 14) of *Sphaerothecum destruens*. A: *Oncorhynchus tshawytscha* (Chinook salmon), B: *O. kisutch* (Coho salmon), C: *O. mykiss* (rainbow trout), D: *Salmo trutta* (brown trout), and E: *S. salar* (Atlantic salmon) The Cyprinidae is represented by seven species belonging to seven genera; F: *Cyprinus carpio* (carp), G: *Rutilus rutilus* (roach), H: *Abramis brama* (bream), J: *Leucaspius delineatus* (sunbleak); K: *Squalius fellowesii.* In the calculation of host specificity, the species *Pseudorabora parva* (topmouth gudgeon; Family Cyprinidae), the species *Oxynoemachelius* sp. (Family Nemacheilidae) and *Lepomis gibbosus* (Family Centrachidae) were also included. The host specificity (STD) was calculated to be 3·82 with a variance of 0·49.

A tree representing the taxonomic hierarchy of *S. destruens* host species was constructed (Fig. 1) using the Linnean classification and the specificity index (STD) for *S. destruens* was calculated to be 3·21 with a variance in taxonomic distinctness (VarSTD) of 0·49; supporting the generalist nature of *S. destruens*.

Using the risk assessment developed by Williams *et al*. (2013) we calculated the potential hazard posed by *S. destruens* to freshwater fisheries in England and Wales with an overall score of 25, identifying this parasite as a high risk parasite. In evaluating the value and susceptibility of native resources, *S. destruens* scored as high risk due to the available evidence indicating that in farmed, semi-natural and controlled exposures *S. destruens* can cause mortality in a number of temperate freshwater species; Atlantic salmon *Salmo salar* (Linnaeus, 1758), and Chinook salmon *Onchorhyncus tshawytscha* (Walbaum)(both in farm conditions and in controlled exposures in the laboratory (Harrell *et al*. 1986; Hedrick *et al*. 1989; Arkush *et al*. 1998), cyprinid species *Abramis brama* (Linnaeus, 1758), *Rutilus rutilus* (Linnaeus, 1758) and *Cyprinus carpio* (Linnaeus, 1758) (controlled exposures, Andreou *et al*. (2012), sunbleak *Leucaspius delineatus* (semi-natural experiments (Gozlan *et al*. 2005) and controlled exposure (Paley *et al*. 2012). The economic and ecological value of freshwater species found in the England and Wales that can be exposed to *S. destruens* (*S. salar, A. brama, R. rutilus* and *C. carpio*) is high as they are key angling species. Fishing rights for salmon robs had an estimated value of £128 million in England and Wales in 2001 and Inland recreational fisheries for the UK had an estimated value of £3 billion in 2001 with carp being the most stocked fish in coarse fisheries, followed by roach and bream being the 4th most stocked (Environment Agency, 2004). In addition, all species inhabit aquatic environments throughout Britain and are key components of lake and river communities. None of the before mentioned susceptible species are threatened in the UK, however, due to the high generalist nature of *S. destruens* we cannot exclude the possibility that additional species from different fish families could also be susceptible; hence only a certainty score of 2 for question 3 (Table 1).

**Table 1.** Risk assessment to determine the hazard risk associated with *Sphaerothecum destruens*

Risk query	Score		Rationale
*A. Value/susceptibility of native resources*	Scorer 1	Scorer 2	
1. What is the economic value of the parasite's host(s) to freshwater fisheries?	4 (3)	3 (3)	*Sphaerothecum destruens* has a wide host range including economically and ecologically important species for fisheries and fish farms; *Salmo salar* (Paley *et al.* 2012), *Abramis brama, Cyprinus carpio, Rutilus rutilus* (Andreou *et al.* 2012). All of these species are of high economic value (Environment Agency, 2004) and inhabit aquatic environments throughout Britain and are key components of lakes and river communities. None of these species are currently threatened or protected in England and Wales (Williams *et al.* 2013). Scorer 2 has scored question 3 with a value of 2 as *Leucaspius delineatus* is endangered in continental Europe and has been found to be highly susceptible to *S. destruens* in the UK
2. What is the ecological value of the parasite's host(s) to freshwater fisheries?	3 (2)	3 (3)	
3. Does the parasite infect a host that is endangered or threatened?	0 (2)	2 (3)	
*B. Colonisation potential*			
4. Based upon climatic conditions of source and recipient localities (including those expected through climate change), what is the likelihood that the parasite will become established?	4 (3)	3 (3)	The colonization potential is high due to its low host specificity and direct lifecycle (Mendonca and Arkush, 2004). Low host specificity – *S. salar, Oncorhynchus tshawytscha, S. trutta, O. mykiss, Salvelinus fontinalis* (Hedrick *et al.* 1989; Arkush *et al.* 1998) *A. brama, C. carpio, L. delineatus* (Andreou *et al.* 2012). Presence of temperature tolerant lifestages (spores and zoospores) and environmental persistence through prolonged release (Andreou *et al*. 2009) increase colonization potential.
5. Based upon the life-cycle development, host specificity and reproductive potential of the parasite, what is the likelihood of successful colonization and spread?	4 (3)	3 (3)	*Sphaerothecum destruens* is a generalist parasite, with low host specificity and a direct lifecycle. It is also closely associated with *Pseudorasbora parva* (Spikmans *et al.* 2013) which has been invasive to England and Wales (Gozlan *et al.* 2010). *Sphaerothecum destruens* could be introduced to new fish farms through *P. parva* and to adjacent wild fish communities through the release of infectious spores.
6. How many legal fish movements take place annually within the risk assessment area comprising susceptible hosts? (0–10 = very low; 10–50 = low; 50–250 = medium; 250–500 = high, >500 = very high).	3 (3)	2 (1)	There is a high risk of parasite spread with fish movement activity; for *A. brama* there are approximately 450 movements recorded annually in England and Wales (Williams *et al.* 2013).
*C. Potential disease risk*			
7. What is the likely pathogenicity of the parasite to fish populations based on disease occurrence in other geographical regions?	3 (2)	3 (3)	Losses have been recorded from aquaculture facilities of Chinook and Atlantic salmon (*O. tshawytscha, S. salar*) (Harrell *et al*. 1986; Hedrick *et al.* 1989). Population declines due to *S. destruens* have been reported in Turkey for several fish species *Oxynoemacheilus* sp. not yet described, *Petroleuciscus smyrnaeus, Squalius fellowesii* (Ercan *et al.* 2015).
8. What is the likely pathogenic importance of the parasite to fisheries based in pathological descriptions and host level changes?	2 (1)	3 (2)	Damage at host level is well studied (Arkush *et al.* 1998). Pathological changes in cyprinids have been described (Andreou *et al.* 2011; Ercan *et al.* 2015) and can in some cases be severe. Scorer 1 has placed a low certainty due to the pathology not being very prevalent in some cases and the difficulty of sampling moribund fish in the wild. In the laboratory however, moribund fish showed severe pathology and behavioural changes – Gozlan *et al.* 2005 for *L. delineatus*.
9. What is the potential disease risk based on the pathogenicity of congeners of the parasite?	2 (1)	3 (2)	*Amphibiocystidium ranae* is a close relative, which has caused high mortalities in frogs (Pascolini *et al*. 2003).
Total	25 (20)	25 (23)	

England and Wales have been used as a case study and thus all questions are answered in relation to native population in England and Wales. The risk assessment follows the guidelines by Williams *et al.* (2013). Both authors filled in the assessment independently and both scores are presented with combined rationales. Scoring criteria are: 0 = very low or no; 1 = low; 2 = moderate; 3 = high; 4 = very high or yes. Certainty scores are provided for every answer, 1 = low, 2 = moderate, 3 = high. Scores were summed and an overall hazard score was calculated. The overall score was then translated to low (0–12 points), moderate (13–24 points) and high (25–36 points) disease risk to native populations. Overall certainty scores were translated as low (1–9 points), moderate (10–18 points) and high (19–27 points).

The colonisation potential of *S. destruens* was also evaluated as high, due to its close association with *P. parva* and its direct life-cycle and environmental transmission (through spores and zoospores). In addition the high fish movements of *A. brama, R. rutilus* and *C. carpio* increase the probability of spreading the parasite to new fish farms and fishing clubs. In 2002/03 the Environment Agency issued consents to stock in excess of 7·5 million fish of which 25% were carp and variants, 8% were for roach and 4% were for bream (Environment Agency, 2004). The close association of these still water bodies with rivers and streams increases the possibility of the parasite being introduced and becoming established in adjacent stream and river communities, through environmental transmission.

The potential of disease risk was scored as moderate to high due to the limited records of population declines linked to *S. destruens* in England and Wales. It is important here to note that evidence provided by the study of Ercan *et al*. (2015) and chronic mortality patterns observed in controlled exposures (Andreou *et al*. 2012) indicate that only long term monitoring of communities potentially in contact with *S. destruens* can detect such population declines. This uncertainty in the lack of evidence was reflected in the low certainty score for this section (mean = 1·8).

**Fig. 2.**
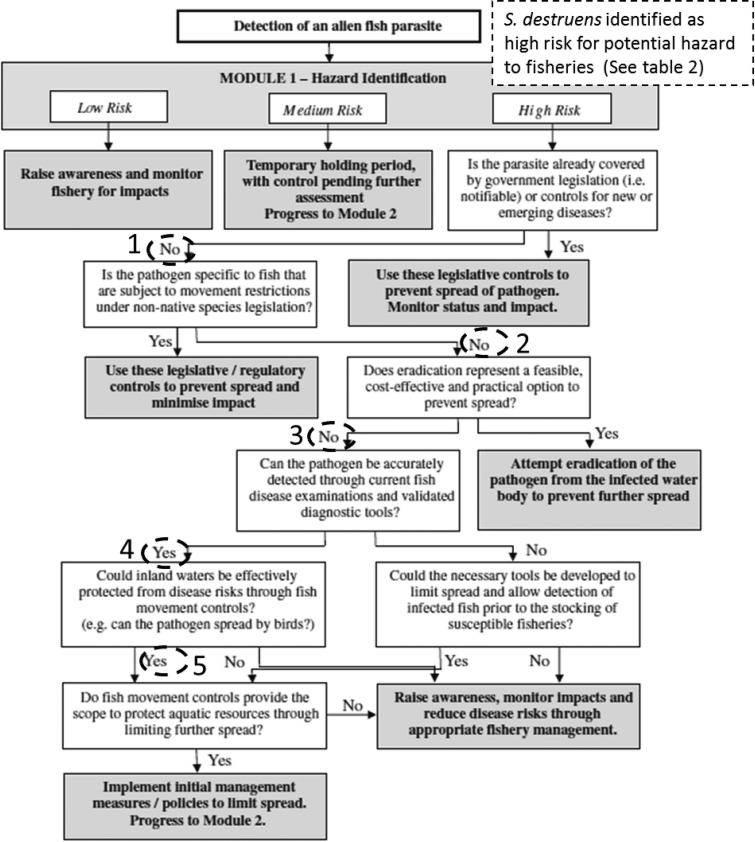
Risk assessment to determine whether management options to control the spread of *Sphaerothecum destruens*. The decision diagram has been adapted from Williams *et al.* (2013). The risk assessment follows the potential hazard assessment posed by the parasite. Refer to the section Results for the rationale supporting the decision made at each step and to Williams *et al.* (2013) for module 2.

Following the hazard risk assessment, the potential management responses were investigated using module 3 from the risk assessment by Williams *et al*. (2013). This assessment accounts for the local/ national legislation and management practices that are already in place as well as the distribution and management policies of the reservoir host (*P. parva*) in the risk assessment area, as that will drive a great part of the risk. The rationale supporting the decision made at each step (Fig. 2) for the case study of England and Wales: (1) *S. destruens* is not currently covered by any legislation in the UK; (2) *S. destruens* can infect *S. salar, A. brama, R. rutilus* and *C. carpio* whose movement is not restricted under the national exotic fish legislation; (3) eradication of the parasite has been attempted by eradicating its healthy host *P. parva* using rotenone a process that is both ecologically and financially expensive and ineffective in removing *S. destruens* from adjacent communities; (4) the parasite can be detected using histology and molecular techniques; (5) fish movement restrictions would be the only effective method to prevent the spread of the parasite to naïve fisheries (however this would depend on the distribution of the parasite, which is currently not known). The recommended management option was to implement initial management measures to limit spread and assess their effectiveness and management of the parasite after these measures are implemented in a panel of experts (as described in module 2 in Williams *et al*. 2013).

## DISCUSSION

### *Sphaerothecum destruens* as a multi-host parasite: implications for disease emergence

Host specificity and cellular tropism is one of the most important characteristics in a parasite's life cycle (Poulin, 2007). Host specificity can be quantified by enumerating the number of species a parasite can infect (Lymbery, 1989). However, this does not provide information on the taxonomic distinctness of the species a parasite can infect. Here *S. destruens*’ observed host specificity is similar to helminths parasitic to Canadian freshwater fish (Poulin and Mouillot, 2003). The high variance around the STD index indicated that *S. destruens* is more likely to colonize new species and in doing so it is possible for the parasite to make bigger taxonomic jumps. Overall, *S. destruens* did not appear to be limited to a phylogenetically narrow host spectrum and the current data suggest that it is a true generalist. In addition, the lack of correlation between genetic distance and susceptibility (Andreou *et al*. 2012) suggests that susceptibility is not dictated by phylogenetic distance increasing the risk of infections to novel hosts cohabiting with species infected with *S. destruens*. It is possible that by exploiting a broader phylogenetic range of hosts, the parasite will use a number of locally available hosts and in doing so will maximize its survival and range expansion opportunities (Krasnov *et al*. 2008). This appears to be a key life history trait of *S. destruens* that could contribute to the parasite's persistence and one that is shared by other Rhinosporideacae members. With the exception of *R. seeberi* (which can infect species belonging to different classes), *S. destruens* is the only Rhinosporideacae member, which can infect species across families. Similar life strategies have been reported for other generalist parasites, notably *Sarcocystis neurona,* which is the cause of equine protozoal myeloencephalitis in horses (Elsheikha, 2009). *Sphaerothecum destruens*’ association with the highly invasive *P. parva* further increases the possibility for range expansion by this parasite and its generalist nature and the high mortalities it can cause in both salmonid and cyprinid species place it as a high risk parasite for freshwater biodiversity.

### Management implications

Parasites such as *S. destruens* cause chronic mortalities, which are extremely difficult to detect in the wild (Gozlan, 2012) in the absence of long term monitoring as evidenced in Ercan *et al*. (2015). Under the Habitats Directive, the health of riverine fish populations is assessed every 5 years, which does not allow the regular monitoring of fish populations to identify and respond to observed declines (European Topic Centre on Biological Diversity, Eionet). Although, bringing together the national fish population assessments in theory is possible, in many cases a combination of missing data or incompatible data (e.g. population sizes reported in different units) makes this exercise impossible. In such cases, the Eionet assessments of conservation status are determined as the proportion of the species in each country and then evaluated. With such approach, it is clear that the emergence of *S. destruens* associated with *P. parva*'s invasion at a site or even catchment level would go undetected. A close look at the latest EU Eionet monitoring, revealed that among *S. destruens* susceptible hosts, only *S. salar* was included and that the great majority of reported data originated from regions such as Scandinavia or Scotland, where *P. parva* is absent.

A good example of the limitation of these types of large scale, weak and uncoordinated monitoring for the purpose of disease emergence, is characterized by the population crash in Europe of the sunbleak *Leucaspius delineates*, one of the most susceptible host to *S. destruens.* It is a species that since *P. parva*'s introduction has become extinct in several European countries and in others has experienced severe population declines. However, it is still not recorded on the Eionet's conservation list, most likely due to data deficiency in Member States assessments.

A number of recommendations are made to policy makers both at the European level and a local level. The potential risk posed by *S. destruens* needs to be urgently re-evaluated in light of the results presented here and an extensive epidemiological survey should be performed by primarily focusing on aquaculture facilities and fisheries where *P. parva* have been reported. *Pseudorasbora parva* need to be screened for infection with *S. destruens* by following a specific sampling strategy involving the sampling of a minimum of 30 fish and the use of molecular techniques to detect the parasite using at least the kidney and liver as the organs of choice. Samples for both molecular analysis and histology should be collected for individual fish. Polymerase chain reaction (PCR) (nested or quantitative PCR (qPCR)) should be used as the first step of detection, followed by histological analysis of fish determined positive by PCR (described in Andreou *et al*. 2012). Where *S. destruens* is detected, wild populations in adjacent water bodies should also be tested for *S. destruens*. This will allow a more informed evaluation of the possibility that *S. destruens* spread can be controlled through fish movement restrictions and inform decisions on restricting fish movement.

### Perspectives

The epidemiology of *S. destruens* in Europe needs to be further investigated, although the current close association of *P. parva* and *S. destruens* (Gozlan *et al*. 2005; Spikmans *et al*. 2013; Ercan *et al*. 2015) suggests that the parasite has been introduced to Europe via *P. parva* invasion. This should be complemented by an extensive review of the literature including technical reports on the health of wild populations that have been cohabited or are in adjacent connected water bodies to *P. parva*. The combination of the parasite's epidemiology as well as any population declines reported in the literature will better inform policy makers on the impact of the parasite as well as on management options. In addition, collection of *S. destruens* positive samples from the wild would further the characterization of *S. destruens*’ invasive status in Europe. The internal transcribed spacer (ITS) region can be used to determine geographic isolation between *S. destruens* populations (Gozlan *et al*. 2009). The existing predictions on host–parasite interactions of generalist parasites suggest that the local diversity of susceptible host community can influence their virulence (Woolhouse *et al*. 2001), which raises concerns for the conservation of fish diversity in Europe.
